# TCP: a tool for designing chimera proteins based on the tertiary structure information

**DOI:** 10.1186/1471-2105-10-9

**Published:** 2009-01-07

**Authors:** Takashi Yoneya, Reina Nishida

**Affiliations:** 1 Research Planning and Administration Department (Tokyo), Research Division, Kyowa Hakko Kirin Co., Ltd., 3 Miyahara, Gunma, 370-1295, Japan

## Abstract

**Background:**

Chimera proteins are widely used for the analysis of the protein-protein interaction region. One of the major issues is the epitope analysis of the monoclonal antibody. In the analysis, a continuous portion of an antigen is sequentially substituted into a different sequence. This method works well for an antibody recognizing a linear epitope, but not for that recognizing a discontinuous epitope. Although the designing the chimera proteins based on the tertiary structure information is required in such situations, there is no appropriate tool so far.

**Results:**

In light of the problem, we developed a tool named TCP (standing for a Tool for designing Chimera Proteins), which extracts some sets of mutually orthogonal cutting surfaces for designing chimera proteins using a genetic algorithm. TCP can also incorporate and consider the solvent accessible surface area information calculated by a DSSP program. The test results of our method indicate that the TCP is robust and applicable to various shapes of proteins.

**Conclusion:**

We developed TCP, a tool for designing chimera proteins based on the tertiary structure information. TCP is robust and possesses several favourable features, and we believe it is a useful tool for designing chimera proteins. TCP is freely available as an additional file of this manuscript for academic and non-profit organization.

## Background

Chimera proteins are widely used for the analysis of the protein-protein interaction region. One of the major issues is the epitope analysis of the monoclonal antibody, and there are conventional methods for the analysis. The prediction of the epitopes in silico is convenient and various algorithms are developed so far. Most of these programs use primary sequence information and properties of amino acid residues [1-6], and therefore, they are applicable to the prediction of the continuous linear epitopes. Recently, Moreau *et al*. proposed the PEPOP which searches the candidates of peptide antigens using tertiary structure information [7]. Many useful antibodies recognize tertiary structures, i.e. the discontinuous epitopes, and algorithms which predict the discontinuous ones are required. To address the problem, some algorithms, e.g. CEP [8], DiscoTope [9] and PEPITO [10], are developed. On the other hand, there are also experimental methods for the epitope analysis, e.g. parallel peptide syntheses [11] and peptide arrays [12]. A well-established method is a phage display [13,14]. This method uses a large size of a peptide library which is presented on a phage protein. The phage clones in the library which have high affinities for the antibody of interest are selected and concentrated iteratively by a so-called biopanning process. After that, the obtained peptide sequences are analyzed, and several methods for the data analysis are developed so far [15-20]. Another common method is the use of chimera proteins [21-23]. Lekcharoensuk *et al*. used the chimera proteins of the type 2 (PCV2) and the type 1 (PCV1) porcine circovirus capsid protein to determine the epitopes for the monoclonal antibodies for the PCV2 [22], and Schoolmeester *et al*. used the human-mouse chimera proteins of integrin α_2 _I-domain for the anti-human integrin α_2 _I-domain antibody [23]. Although they used only primary sequence information, Karisola *et al*. used the tertiary structure information to design the hevein-AMP chimera proteins for the epitope mapping of an allergen [21]. A typical example of how to use of the chimera proteins is described below. For example, an antibody assumes to recognize a human protein, but not to recognize the mouse orthologue. In this situation, a continuous portion of the human antigen is substituted by the corresponding mouse sequence, and then, the binding activity to the chimera antigen is analyzed. If the substitution does not affect, an additional region is substituted again. The epitope region is narrowed down by repeating the process. This method works well for continuous epitopes, but can not narrow down the epitope region enough in case of the discontinuous ones. Although the chimera proteins should be designed based on the 3D structure information for the monoclonal antibodies which recognize discontinuous epitopes, there is no such a tool so far.

In light of this problem, we developed a tool named TCP (standing for a Tool for designing Chimera Proteins) for designing chimera proteins based on the tertiary structure information. It extracts some sets of three mutually orthogonal cutting surfaces (CSs) for designing chimera proteins. We believe this algorithm should be a useful tool for preparing chimera proteins.

## Methods

### Exploration of CSs with a genetic algorithm

The purpose of this program is the extraction of several sets of three mutually orthogonal CSs which divide the target region (TR), e.g. a whole polypeptide or a particular domain, into two parts. An overview of the algorithm for searching the CSs is described here. First, the centroid of the TR's alpha carbons (CAs) is calculated using the coordinates in a protein data bank(PDB) [24,25] file and appropriate numbers of the first normal vectors for random directions, but the coordinate values are integers between 0 and 63, are generated. Next, a second vector which is orthogonal to the first vector is generated and the vector is rotated by appropriate angles. Then, third vectors which are orthogonal to the first and the second vectors are generated. Through the process, a series of the second and the third vectors are obtained for each first vector, and the three vectors are mutually orthogonal. The TR is divided into eight portions by a set of the three CSs containing the centroid defined by the three normal vectors. A reasonable criterion of a good CS set is that the set divides the TR into even parts. Therefore, we select a pair of the second and the third vectors which most evenly divide the TR along with the first vector using the variances of the number of residues in the eight portions. Practically, a percentage of the coefficient of variation (CV) is used instead of the variance, and the CV is defined as the ratio of the standard deviation to the average.

Next, the generated normal vectors and centroids are optimized by the genetic algorithm (GA) which is a well-known heuristic method to find exact or approximate solutions [26]. The first normal vector is encoded as three 6-bit-binary strings and a single recombination is introduced to the fixed number of parents, and the coordinates of the centroids are swapped between the parents. After the recombination, mutations are introduced to all genes except for the individual having highest fitness value at the 5% of the mutation rate. After the binary to decimal conversion, the genes are mutated with random number within appropriate ranges. The mutation with random numbers is also subjected to the coordinates of the centroid. The ranges are 10 for normal vectors and 1Å for the centroid at the 5% of the mutation rate. After the manipulations, the individuals are sorted by the fitness values and fixed population is selected. The inverse of the CV of the number of residues in the eight portions is used as the fitness function and maximized. This process is repeated for proper generations or until satisfying a stopping condition. To avoid the local optima, the whole process is repeated several times.

### Selection of unique CS sets

Although the CS sets are selected based on the even separation of the TR, a user would select the suitable CS sets with more information, e.g. the comparison with the orthologue, the solvent accessible surface area (ASA) etc. Therefore, it is better to present several candidates with different patterns. Here, we define a value, *S*_*i*,*n*_, which is 1 in case that the *n*-th CA (*CA*_*n*_) is located on the plane *i *(*P*_*i*_) or at the forward side of *P*_*i*_, and -1 in case that the *CA*_*n *_is located at the backward side. The direction is specified by the normal vector of *P*_*i*_. Using *S*_*i*,*n*_, the TR is divided into two parts. The number of the target residues is denoted as *N*, and the pattern vector, *M*_*i*_, is defined as follows.

$$M_{i}=\left(\frac{S_{i,1}}{\sqrt{N}},\frac{S_{i,2}}{\sqrt{N}},\frac{S_{i,3}}{\sqrt{N}},...,\frac{S_{i,N}}{\sqrt{N}}\right)$$

After dividing the TR, the divided pattern with the plane is evaluated whether it is similar to that by already selected planes. To evaluate the similarity, a function, *Sim*, is defined as follows.

$$Sim(P_{i},P_{j})=\left|M_{i}\cdot M_{j}\right|=\left|\frac{1}{N}\sum_{n=1}^{N}\left(S_{i,n}S_{j,n}\right)\right|$$

**Figure 1:**
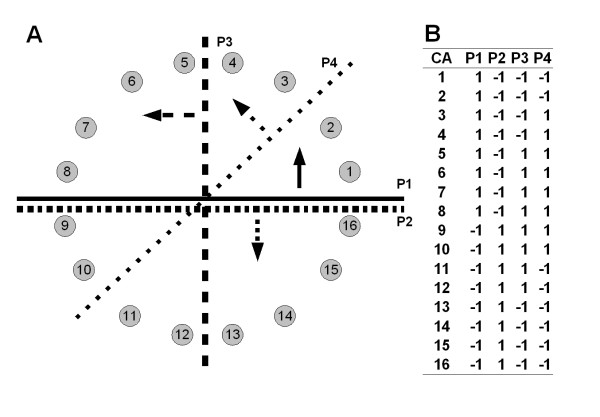
**The *Sim *score between two CSs**. It is presumed that a TR has 16 CAs and they are randomly distributed around the centroid (A). The TR is divided by four CSs (P_1_, P_2_, P_3 _and P_4_) defined by the indicated normal vectors and including the centroid. The *S*_*i*,*j *_values of the CAs are shown in (B). If the TR is divided by two planes and the angle between the normal vectors is zero, i.e. P_1_and P_1_, the *Sim *score is *Sim(P*_1_,*P*_1_) = |-16/16| = 1. Similarly, if the angles between the vectors are π (i.e. P_1 _and P_2_), π/2 (i.e. P_1 _and P_3_) and π/4 (i.e. P_1 _and P_4_), the *Sim *scores are *Sim(P*_1_,*P*_2_) = |-16/16| = 1, *Sim(P*_1_,*P*_3_) = |0/16| = 0 and *Sim(P*_1_,*P*_4_) = |8/16| = 0.5, respectively.

*M*_*i*_·*M*_*j *_is the dot product of *M*_*i *_and *M*_*j*_. As shown in Figure 1, the more different the separating patterns are, the *Sim(M*_*i*_, *M*_*j*_) value approaches zero. Based on the score, the newly created plane is evaluated whether it is different from the already selected ones or not. Although the concept is explained with a single plane case, the pattern vector is expanded for the combination of three planes. All of the CS sets which are generated in the selection process are sorted by the fitness values and the *Sim *score is evaluated from top to bottom. If the *Sim *score is less than a threshold, the CS set is selected as a unique one.

## Implementation

**Figure 2:**
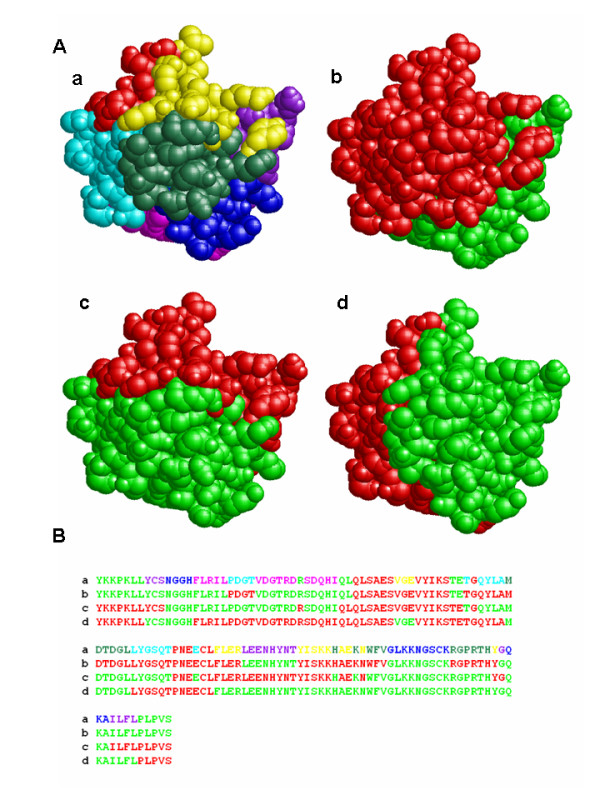
**The colour-coded structures of human FGF1**. The colour-coded tertiary structures (A) and the primary sequences (B) of 1EVT:A divided by three CSs. The colour relationship between the eight and the two portions is summarized in Table 1. The numbers of residues in the eight and the two parts are shown in Table 2 and Table 3, respectively.

**Figure 3:**
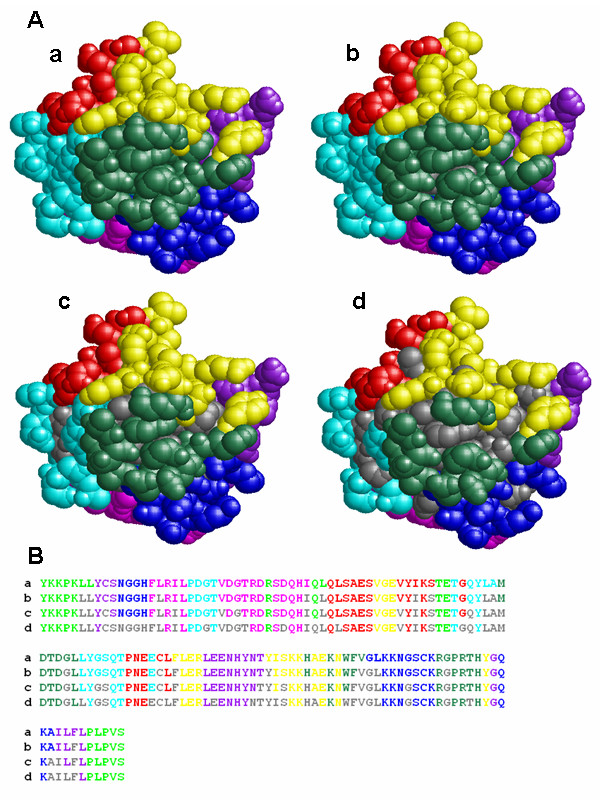
**The use of the solvent accessible surface area (ASA)**. The colour-coded tertiary structures (A) and the primary sequences (B) of 1EVT:A divided with a set of three CSs. The residues having the smaller ASA than the thresholds are coloured grey. The ASAs are calculated by DSSP. The result of DSSP is not incorporated in a. The thresholds of the ASAs in b, c and d are 0, 10 and 30 Å^2 ^respectively.

**Figure 4:**
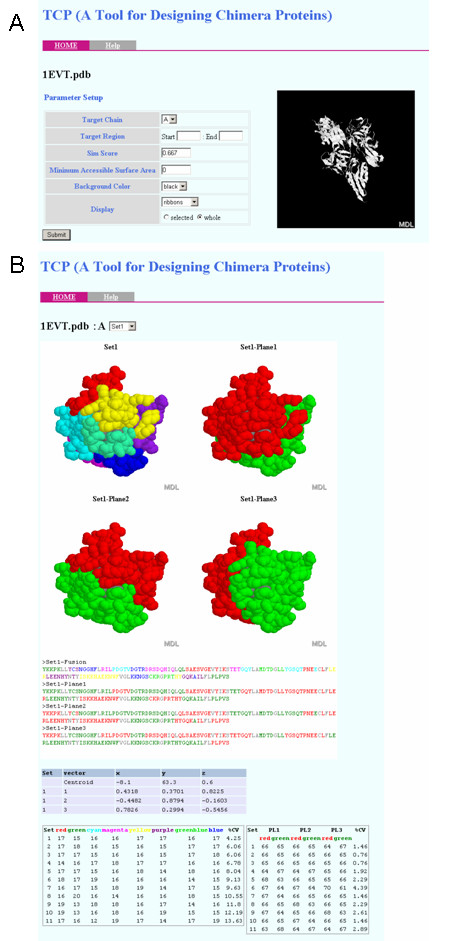
**The screenshots of the web interface**. The parameter setup screen (A) and the resultant colour-coded structures (B). At first, a PDB file and a DSSP file (option) are uploaded to the server. TCP parses the PDB file and the DSSP file, and displays the parameter setup menu (A). A user selects the target chain, the target region (TR), etc., and submits the parameters. TCP analyzes the TR and displays the result in the web-browser (B). The upper part shows the colour-coded 3D structures and the primary sequences. By using the web interface, four figures of the TR divided by the combination of the three CSs and each CS can be seen at once. The lower part shows the centroids and the normal vectors defining the CSs, and the numbers of residues in each part. The set of the CSs can be changed by the pull-down menu. In this example, the target is 1EVT:A and the threshold of the ASA is 0 Å^2^.

TCP was implemented with Perl as a set of three command-line programs, and outputs the colour-coded sequences as a rich text file and a set of script files for RasMol [27,28], which is one of the most popular 3D molecular graphics viewers, to display the colour-coded tertiary structures (Figure 2, Table 1). In the colour-coding process, the result of a DSSP program [29] can be incorporated. DSSP is a popular program defining the secondary structures and the ASA from the atomic coordinates in a PDB file. TCP shows the buried residues in grey based on a threshold of the ASA calculated by DSSP (Figure 3). Although the execution time depends on the TR and parameters, it takes a few minutes under the default condition with a standard PC, such as a 2.4 GHz Pentium 4 processor with a 512 MB RAM. The web interface is also developed and it is coded with PHP and runs on Linux and Apache. The colour-coded structures are displayed on a browser with a Chime plug-in [30] (Figure 4).

**Table 1:** The colour relationship between the combination of the three CSs and each CS

**Fusion**	**CS1**	**CS2**	**CS3**
red	red	red	red
green	green	red	red
cyan	red	green	red
magenta	green	green	red
yellow	red	red	green
purple	green	red	green
greenblue	red	green	green
blue	green	green	green

Fusion, CS1, CS2 and CS3 correspond to a, b, c and d of Figure 2, respectively

## Results

### Exploration of the CS sets with five proteins

**Figure 5:**
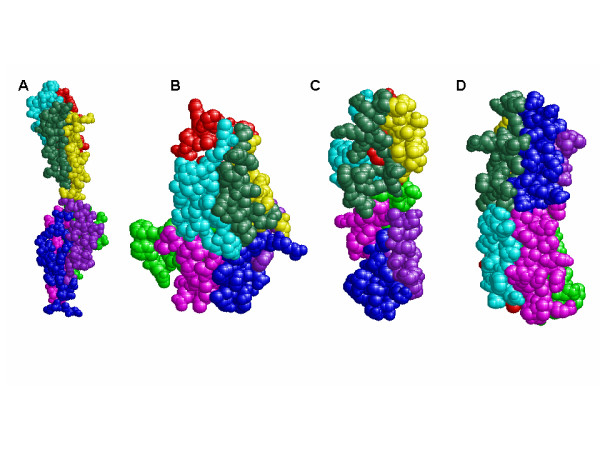
**The colour-coded tertiary structures of human FGFR1, human TNF-beta, human TNFR-p55 and an ankylin domain of human Notch1**. A: human FGFR1 (1EVT:C), B: human TNF-beta (1TNR:A), C: human TNFR-p55 (1TNR:R), D: an ankylin domain of human Notch1 (1YYH:A). The numbers of the residues in the eight portions are shown in Table 2.

At first, human FGF1 was used to test our algorithm and the PDB identifier is 1EVT. FGF1 is a well-known globular protein which was originally isolated as a stimulation factor of fibroblasts [31]. Hereinafter, the polypeptide is specified as "PDB identifier:Chain". For example, the notation 1EVT:A means the chain A of 1EVT. Our algorithm found sets of three mutually orthogonal CSs and the colour-coded figures using one of the CS sets are shown in Figure 2. This result indicates that the TR was almost equally divided into two portions by each CS, and into eight by the combination of the three CSs. To evaluate whether our algorithm is applicable to various shapes of proteins, it was tested with four other polypeptides, 1EVT:C, 1TNR:A, 1TNR:R and 1YYH:A. As shown in Figure 5, Table 2 and Table 3, TCP found sets of three CSs which divided the TRs almost equally into two portions by each CS and into eight by the combinations. It is noteworthy that 1EVT:C and 1TNR:R were almost equally divided in spite of the elongated shapes. Therefore, TCP should be applicable to various shapes of proteins. The numbers of residues in the divided portions of 1EVT:A and 1TNR:R are listed in Table 4. Although the 1EVT:A was almost equally divided into eight portions by most of the CS sets, the 1TNR:R was almost equally divided by a CS set. It indicates that the equal partition of the TR is not a common result even if the TRs are divided by the mutually orthogonal three CSs, and the number of the CS sets which divide the TR equally into eight parts highly depends on the structures of the TRs.

**Table 2:** The numbers of residues divided into eight portions by a combination of three CSs

**Chain**	**R**	**G**	**C**	**M**	**Y**	**P**	**GB**	**B**	**%CV**
1EVT:A	17	17	16	16	16	16	17	16	2.96
1EVT:C	21	25	22	25	23	26	23	26	7.39
1TNR:A	17	19	19	17	18	18	18	18	3.93
1TNR:R	18	18	17	17	16	17	18	18	4.01
1YYH:A	24	24	25	25	25	23	23	24	3.24

The colour labels, R, G, C, M, Y, P, GB and B, represent red, green, cyan, magenta, yellow, purple, greenblue and blue, respectively. The values in the row labelled with 1EVT:A are the actual counts of the residues shown in

**Table 3:** The others are the actual counts of them shown in Figure 5. Table 3 – The numbers of residues divided into two portions with three CSs

**Chain**	**CS1**		**CS2**		**CS3**	
	
	**R**	**G**	**R**	**G**	**R**	**G**
1EVT:A	66	65	66	65	66	65
1EVT:C	89	102	95	96	93	98
1TNR:A	72	72	72	72	72	72
1TNR:R	69	70	69	70	70	69
1YYH:A	97	96	96	97	98	95

The colour labels, R and G, represent red and green, respectively. The values in the row labelled with 1EVT:A are the actual counts of the residues shown in Figure 2. The others are the actual counts of them shown in Figure 5.

**Table 4:** The partition of 1EVT:A and 1TNR:R with 11 sets of three CSs

**Set**	**1EVT:A**									**1TNR:R**								
	
	**R**	**G**	**C**	**M**	**Y**	**P**	**GB**	**B**	**%CV**	**R**	**G**	**C**	**M**	**Y**	**P**	**GB**	**B**	**%CV**
1	17	17	16	16	16	16	17	16	3.0	18	18	17	17	16	17	18	18	4.0
2	17	17	15	16	16	15	17	18	6.1	17	25	10	19	22	7	21	18	32.7
3	15	17	18	16	17	17	16	15	6.1	7	26	12	26	27	11	26	4	52.7
4	16	18	18	15	17	15	16	16	6.8	16	24	30	2	4	28	22	13	56.6
5	18	16	16	17	16	15	15	18	6.8	31	5	28	16	1	25	8	25	61.9
6	19	16	14	18	17	15	16	16	9.1	26	1	14	30	22	4	8	34	66.8
7	18	17	15	15	15	15	17	19	9.1	6	25	27	12	5	31	32	1	68.4
8	17	19	15	15	14	16	18	17	9.6	30	16	35	3	3	26	1	25	73.0
9	20	15	18	15	15	16	16	16	10.1	30	8	25	5	8	27	0	36	73.0
10	16	15	16	17	16	20	14	17	10.1	31	12	6	30	29	0	1	30	75.3
11	15	16	16	19	18	18	13	16	11.0	10	27	8	33	33	0	28	0	77.2

This table shows the numbers of the residues in the eight portions and the %CV of 1EVT:A and 1TNR:R. The colour representation is described in Table 2.

### The performance comparison between the GA-based method and the random search

The performance of the GA-based method was compared with a simple random search (RA) method. The %CV values of the eight portions divided by the best CS sets and the execution times are shown in Table 5. The number of the generating CS sets in the RA-based method was adjusted with the maximum number of the CS sets in the GA-based method. As shown in Table 5, the GA-based method found the better CS sets for all polypeptides in a short time, and therefore, it overcame the RA-based method.

**Table 5:** A performance comparison of the genetic algorithm and the random search

**Chain**	**GA**		**Random**	
	
	**%CV**	**Time**	**%CV**	**Time**
1EVT:A	3.8	85.4	3.9	239.1
1EVT:C	8.2	168.7	9.2	353.7
1TNR:A	3.6	137.1	8.1	264.2
1TNR:R	3.3	137.3	8.2	254.4
1YYH:A	2.2	167.7	3.6	344.2

Average	4.2	139.2	6.6	291.1

The %CV of the eight portions divided by the best CS set and the execution times (seconds) are shown. The tests were repeated ten times by a personal computer with a 2.4 GHz Celeron D processor and 1,024 Mb RAM, and the results were averaged.

### The relationship between the Sim score and the number of the selected CS sets

We also tested how many CS sets were selected on the five proteins with various thresholds. As shown in Figure 1, the rough idea of the angle is obtained from the thresholds by assuming the random distribution of the CAs. The five thresholds, 0.01, 0.333, 0.5, 0.667 and 0.75, correspond to π/2, π/3, π/4, π/6 and π/8, respectively. Although zero corresponds to π/2, 0.01 was used instead. Because the numbers of the target residues were odd number except for 1TNR:A, the scores of the four proteins were always greater than zero and only one CS set was selected if the thresholds was zero. The test was repeated ten times with the five proteins, and the counts of the selected CS sets were averaged (Table 6). Interestingly, the threshold affected the number of the selected CS sets but the difference of the TR did not. The shapes of the five proteins are various as described so far, and it means that the shapes of the TRs do not affect the number of the selected CS sets. It is a favourable feature because the algorithm is applicable to various shapes of proteins with the same parameter, and a universal parameter giving preferable numbers of the CS sets is adjustable by the *Sim *score.

**Table 6:** The numbers of the selected the CS sets with various *Sim *scores as the thresholds

**Chain**	**Thresholds of the *Sim *score**				
	
	**0.010 **	**0.333 **	**0.500 **	**0.667 **	**0.750 **
1EVT:A	2.0	2.9	5.5	11.5	19.4
1EVT:C	2.0	3.0	4.4	12.0	17.7
1TNR:A	1.5	3.4	5.7	11.3	16.7
1TNR:R	1.4	2.4	4.3	11.0	17.2
1YYH:A	1.1	2.1	3.1	6.6	9.9

Average	1.6	2.8	4.6	10.5	16.2

The tests were repeated ten times with various thresholds of the *Sim *scores, and the results were averaged.

### Analysis of the antigen-antibody complexes

**Figure 6:**
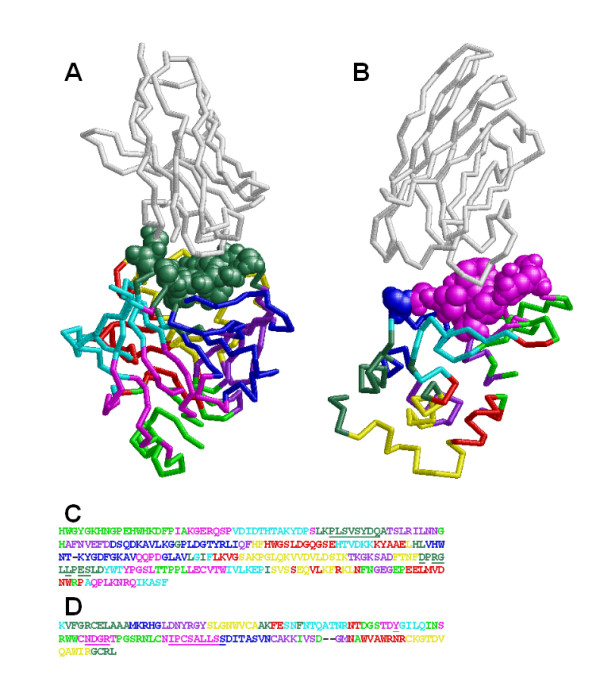
**Analysis of the complexes of the camel antibody heavy chain fragment (cHC) with bovine carbonic anhydrase (bCA) or chicken egg lysozyme (cEL)**. A, B: The complex structures of the cHC with the bCA (A) or the cEL (B). The cHC, 1G6V:K in A and 1ZVH:A in B, are coloured white. The bCA (1G6V:A in A) and the cEL (1ZVH:L in B) are coded by eight colours. The residues whose CAs are located within 8 Å of the CAs of the antibodies are shown by space fill. C, D: The colour-coded amino acid sequences of the bCA (C) and the cEL (D). The residues shown by space fill in A and B are underlined in C and D, respectively.

**Figure 7:**
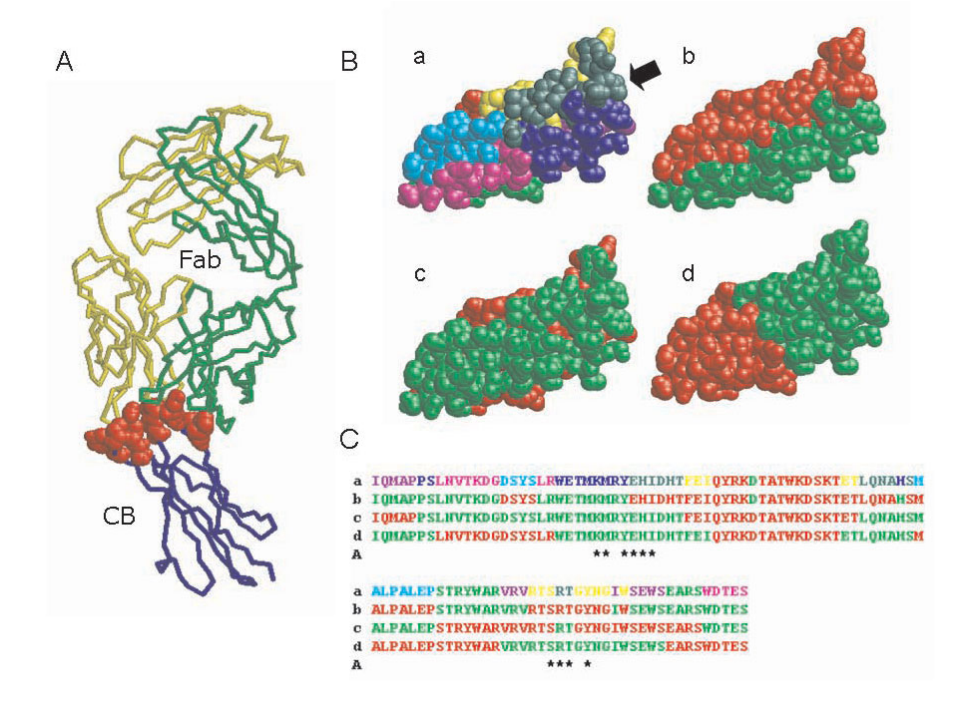
**Analysis of the complex structure of a domain of the GM-CSF receptor common beta chain (CB) and the Fab fragment of the monoclonal antibody**. A: The complex structure of the CB and the Fab fragment. The heavy chain (1EGJ:H) and light chain (1EGJ:L) of the Fab are coloured green and yellow, respectively. The CB (1EGJ:A) is coloured blue and the residues whose CAs are located within 8 Å of the CAs of the Fab are coloured red. B: The colour-coded structure of 1EGJ:A. The black arrow indicates the binding region of the Fab. C: The amino acid sequences of the colour-coded structures shown in B. The asterisks indicate the red-coloured residues in A.

We show some examples analysing the antigen-antibody complexes. First, the complexes of the camel antibody heavy chain fragment (cHC) with the bovine carbonic anhydrase (bCA) or the chicken egg lysozyme (cEL) were analyzed (Figure 6). The residues whose CAs are located within 8 Å of the CAs of the cHC are shown by space fill. The fourteen residues of bCA, which are in the greenblue area, are closely located to the cHC (Figure 6A and 6C). The fourteen residues of the cEL, the thirteen residues of them are in the pink area and a residue of them is in the blue one, are closely located to the cHC (Figure 6B and 6D). Although the greenblue area of the bCA and the pink one of the cEL are matched with the interaction surfaces well, we should note that these are the specific examples and one or two borders are located in the binding areas in the most cases. Next, the complex structure of a domain of the GM-CSF receptor common beta chain (CB) and the Fab fragment of the monoclonal antibody was analyzed. As shown in Figure 7, ten amino acid residues of the CB are closely located to the Fab. These residues disperse to the three parts, greenblue, yellow and blue, in Figure 7 B-a (hereinafter, it is represented as B-a), and the interaction surface includes two borders of the CS sets. In the two-part designs, these residues belong to the green part in the B-d and disperse to the both parts in the B-b and the B-c.

### Description of the software

TCP has two types of the interface, a stand-alone command line program and a web-based server-client system. As shown in Figure 4, TCP displays the colour-coded primary sequences as the FASTA format and the tertiary structure images in the web browser. The command-line program also outputs the results but the primary sequences are written down in a rich-text file and the tertiary structures are output as the RasMol scripts. A benefit of the web-interface is that the tertiary structures of the four designs by a CS set are displayed at once and the selected CS set can be changed by the pull-down menu. Most parameters, i.e. the target chain, the target region, the threshold of the *Sim *score, the threshold of the ASA, the background colour, the style and the region to display the structures, can be set by the user (Figure 4A), and these parameters are also set in the command-line program as the command-line arguments. The changes of the target region and/or the thresholds of the *Sim *score require the exploration of the CS sets, and therefore the execution takes a few minutes. The execution to change the other parameters, i.e. the threshold of the ASA and the display parameters of the tertiary structures, is completed in a moment.

## How to use the results

**Figure 8:**
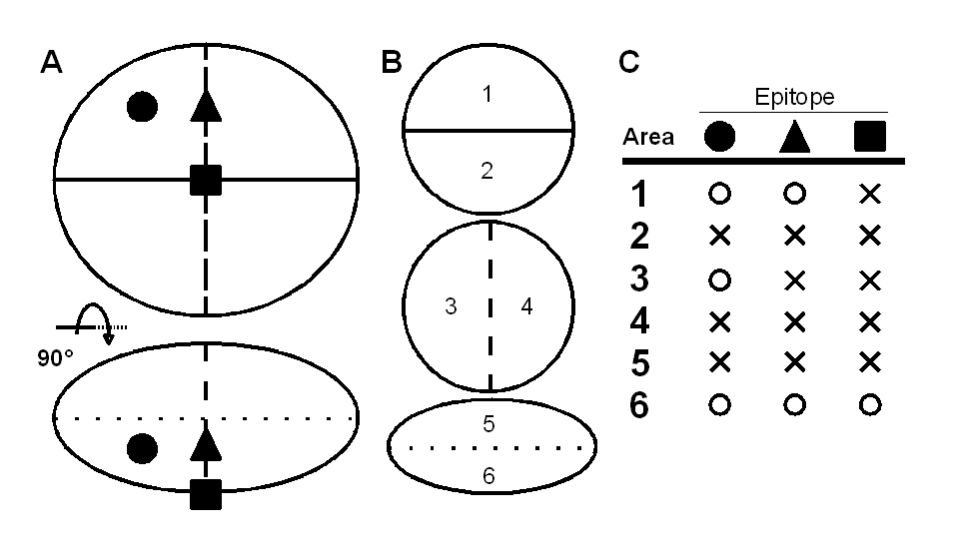
**The use of chimera proteins divided into two portions for epitope analysis**. A: Projection images of a protein from two viewpoints. The lower is the image of that the upper is rotated around the horizontal axis by π/2. Three epitopes are postulated in the protein and indicated by a filled circle, a filled triangle and a filled box. Three CSs are indicated with a solid line, a broken line and a dotted line. B: The six divided portions with the three CSs are labelled with numbers. C: The expected binding pattern of each epitope indicated in A. A portion with the number shown in B is the original sequence and another is modified. For example, the area 1 means that the portion 1 is the original sequence and the portion 2 is the substituted sequence. It is expected that the antibodies which recognize one of the two epitopes labelled as the filled circle or the filled triangle bind the protein labelled as 1, but antibodies which recognize the epitope labelled as the filled box does not.

The TCP outputs two types of designs consisting of two and eight parts. If a set of three designs dividing the TR into two parts are adopted, six kinds of chimera proteins, i.e. two for each design, should be prepared (Figure 8). In this case, the antibody binds three of the six chimera proteins if the antibody does not recognize the borders. If the antibody recognizes one or two borders, the antibody binds two or one chimera proteins, respectively. Based on the binding patterns, the epitope region should be assigned. If a design dividing the TR into eight parts is adopted, two methods are considerable. One is the preparation of the eight chimera proteins which contain an original portion and seven orthologous portions. If an epitope is located on the borders, the antibody binds none of the chimera proteins and therefore, the epitope should not be assigned by this method. Another is the preparation of the eight chimera proteins which contain an orthologous portion and seven original portions. In this case, the obtained information is the same from the three two-part designs, but two more chimera proteins should be prepared. Therefore, we recommend preparing the chimera proteins based on each CS and use of the eight-part design to choose a CS set.

## Discussion

There is no doubt about the usefulness of chimera proteins for epitope analysis [21-23] and a simple tool for the design is required. The usefulness of the chimera proteins was demonstrated not only for the epitope analysis of the monoclonal antibodies [22,23] but also for the common allergen epitopes [21]. We developed TCP as an easy to use program for the purpose and proved the robustness using five real protein structures so far. TCP outputs some sets of four designs, i.e. three two-part designs and an eight-part design. Users could choose the preferred designs based on their purposes and prior information. The application was explained without considering the location of each residue so far. As shown in Figure 3, the residues having small ASAs are coloured grey with various thresholds. The buried residues are less probable to be parts of the epitope. In addition, substitutions of the buried residues may break the tertiary structure. Therefore, it is better to substitute the residues located only at the surface, and the ASA information should be useful for designing the chimera proteins. And then, the information of the sequence conservation is also useful because the conserved residues could be set aside if the antibody does not recognize the orthologue. To use such extra information, the divided patterns by several CS sets should be examined, and therefore, an appropriate threshold of the *Sim *score should be set to reduce the sets to the appropriate numbers.

Although we used not only single domain polypeptides but also multi domain ones for the evaluations, the TR is mostly narrowed down to a single domain by truncations prior to preparing the chimera proteins, and therefore, a single domain is generally used as the TR. The majority of the domains are less than 200 residues [32,33]. If the epitope region is narrowed down until an eighth part of the TR using the chimera proteins designed by TCP, the typical examples are shown in Figure 6, the number of residues in the region is at most 25 residues in many cases. Furthermore, the number of residues becomes smaller if the buried residues are set aside. But in many cases, the epitope regions would include one or two borders, like Figure 7. Even if the epitope is not narrowed down until an eighth part, the information that the epitope locates on the border is obtained. This information is also useful for the estimation of the epitope region. In the case of Figure 7, the monoclonal antibody would bind one of the chimera proteins based on the Figure 7 B-d (hereinafter, it is represented as B-d), and would not bind the both chimera proteins based on the B-b and the B-c. If such results were obtained, it is deduced that the epitope region is located in the green portion of the B-d and on the border of the B-b and the B-c. Because the regions which closely located to the both borders of the B-b and the B-c are quite restricted in the green portion of the B-d, the epitope would be deduced as the quite restricted area. As described with some examples of the antigen-antibody complex, epitopes would be narrowed down quite well in many cases, and we believe the TCP is a useful tool for the analysis of protein-protein interactions.

## Conclusion

We developed TCP, a tool for designing chimera proteins based on the tertiary structure information. As described so far, TCP is robust and possesses several favourable features. We also showed the results of the analysis of three different antibody-antigen complexes. These results should help to imagine the practical use of our program. In conclusion, we believe TCP is a useful tool for many experimental scientists.

## Availability and requirements

TCP is freely available as Additional file 1 of this manuscript for academic and non-profit organizations. It requires Perl and RasMol and runs on Window and Linux as a set of command-line programs. The web interface runs on Linux and requires Apache, Perl and PHP for the server and a Chime plug-in is required for the client.

## Authors' contributions

TY conceived the study, designed and programmed the command-line program, and drafted this manuscript. RN designed and programmed the web interface. All authors read and approved the final manuscript.

## Supplementary Material

Additional file 1 **TCP_package. A complete package of the TCP program**. Click here for file
