# Supplementary material for: TCP: a tool for designing chimera proteins based on the tertiary structure information
Source: BMC Bioinformatics. 2009 Jan 7;10:9. doi: 10.1186/1471-2105-10-9 (PMC2631521; doi:10.1186/1471-2105-10-9)
Supplement: Additional file 1 — TCP_package. A complete package of the TCP program. [file 1471-2105-10-9-S1.zip › TCP_package/help.html]

TCP (A Tool for Designing Chimera Proteins)


# TCP (A Tool for Designing Chimera Proteins)

  

|  |  |  |
| --- | --- | --- |
| HOME | Help |  |

  
  

## Help

  

### Input

- **PDB file**
  

Specify a file from your local disk, in PDB format.  

- **DSSP file (Option)**
  

DSSP is a popular program defining the secondary structures and the solvent surface accessible area (ASA) from atomic coordinates of a PDB file.
You need the file to set the Minimum Accessible Surface Area parameter in a script.
  

### Parameter Setup

- **Target Chain**
  

a target chain in the coordinate file  

- **Target Region**
  

- the start position of the target chain for analysis (option; default: the N-terminus of the chain)
  
- the end position of the target chain for analysis (option; default: the C-terminus of the chain)
  

- **Sim score**
  

a threshold of the maximum Sim score (option; default: 0.667). The detail of the Sim score is written in our paper  

- **Minimum Accessible Surface Area**
  

the minimum solvent accessible surface area to label the residues (option; default: 0)  

- **Background color**
  

change the background color of 3D structure display
- **Display**
  

- a display style in CHIME
  
- display whole chain or selected target region
  

  

### Result

The upper figures are the color-coded 3D structures and the primary sequences. By using the web interface, four figures of the TR divided by the combination of the three cutting surfaces (CSs), CS1, CS2 and CS3 can be seen at once. The lower tables show the numbers of residues in each part.
The set of the CSs can be changed by the pulldown menu.

- **Color relationship**
  
  

| Color | Plane1 | Plane2 | Plane3 |
| --- | --- | --- | --- |
| red | red | red | red |
| green | green | red | red |
| cyan | red | green | red |
| magenta | green | green | red |
| yellow | red | red | green |
| purple | green | red | green |
| greenblue | red | green | green |
| blue | green | green | green |


  
  
  
  
