# Supplementary material for: TCP: a tool for designing chimera proteins based on the tertiary structure information
Source: BMC Bioinformatics. 2009 Jan 7;10:9. doi: 10.1186/1471-2105-10-9 (PMC2631521; doi:10.1186/1471-2105-10-9)
Supplement: Additional file 1 — TCP_package. A complete package of the TCP program. [file 1471-2105-10-9-S1.zip › TCP_package/ReadMeFirst.pdf]

Instructions

List of files

- ReadMeFirst.pdf : this file
- SearchSurface.pl : a script to search the sets of normal vectors
- ChimeraColor.pl: a script to output color-coded sequences and the RasMol scripts
- TCP.pl: a script to run the two scripts at once
- index.php: a php script of the web interface
- help.html: a help file of the web interface
- 1EVT.pdb: a coordinate file of the complex of human FGF1 and the ligand binding domains of human FGFR1
- 1EVT_A.dssp.out: a file treated the coordinate of the chain A of 1EVT.pdb with DSSP

Requirements

The command-line program requires Perl [1, 2] and RasMol [3] and it was confirmed to run on Windows XP and Linux (Fedora Core 5). To use the web interface, Perl, PHP and Apache are required for the server and a Chime plug-in [4] is required for the client. The web interface was confirmed to run on Linux (Fedora Core 5).

Descriptions of the scripts

- SearchSurface.pl
    - Description of the script

      This script creates a working directory, and extracts the coordinates of the specified region from a given PDB file and exports it to a file named target.pdb. Next, it calculates the centroid of the alpha carbons. After that, it searches the sets of three normal vectors for the planes to divide the target region (TR) with genetic algorithm. Finally, it outputs the results to two files named NormalVectorAndCentroid.txt and ColorDistribution.txt. The normal vectors and the coordinates of the centroids are listed in the NormalVectorAndCentroid.txt and the numbers of the residues of the TR are listed in the ColorDistribution.txt.
    - Syntax : perl SearchSurface.pl -p 'pdbfile' -o 'output_directory' -c 'target_chain' [-s 'start' -e 'end' -S 'sim_score' -d 'yes/no']
    - Descriptions of the arguments

      -p: a coordinate file of a target protein in PDB format (essential)

-o: a directory in which the results are output (essential)
-c: a target chain in the coordinate file (essential)
-s: the start position of the target chain for analysis (option; default: the N-terminus of the chain)
-e: the end position of the target chain for analysis (option; default: the C-terminus of the chain)
-S: a threshold of the maximum Sim score (option; default: 0.667). The detail of the Sim score is written in our paper
-d: delete the specified directory if it exists prior to execution (option; default: no)

- ChimeraColor.pl
  - Description of the script
    This script colors the protein sequence based on the sets of three planes. It outputs the color-coded sequences to a rich-text file named Separate.rtf, and a set of the RasMol scripts in a rasmol_scripts directory. The file names of the RasMol scripts correspond to the names of sequences in the Separate.rtf. This script also incorporates the result of the DSSP [5, 6] and colors the sequence based on the solvent accessible surface area.
  - Syntax : perl ChimeraColor.pl -o 'output directory' -c 'target chain' [-D 'dssp file' -a 'minimum accessible surface area' -r 'display style in RasMol'
  - Descriptions of the arguments
    -o: a directory in which the results are output (essential)
    -c: a target chain in the coordinate file (essential)
    -D: the output file of the DSSP (option)
    -a: the minimum solvent accessible surface area to label the residues (option; default: 0 Å^2).
    -r: a display style in the RasMol (option; default: spacefill).
- TCP.pl:
  - Description of the script
    This script runs the two scripts described above.
  - Syntax : perl TCP.pl -p 'pdbfile' -o 'output_directory' -c 'target_chain' [-s 'start' -e 'end' -S 'sim_score' -d 'yes/no' -D 'dssp file' -a 'minimum accessible surface area' -r 'display style in RasMol']
  - Descriptions of the arguments
    The arguments are the same with those as described above.

Tutorial on the command-line program

We make a short tutorial instead of the manual. This tutorial is explained using a Windows PC. Perl and RasMol should be installed properly as instructed by the software. At the beginning, create a directory named "Tutorial" and put the three script files (i.e. TCP.pl, SearchSurface.pl, and ChimeraColor.pl), 1EVT.pdb and 1EVT_A.dssp.out into the directory. Next, start the command line and move to the directory and type as follows.

```
> perl TCP.pl -p 1EVT.pdb -o FGF -c A -d yes
```

A directory named FGF is created, and three files and a directory are created in the FGF directory. The d option permits to delete the target directory if it exists. Open a file named Separate.rtf in the FGF directory.

```
>Set1-Fusion
YKKPKLLYCSNGGHFLRILPDGTVDGTRDRSDQHIQLQLSAESVGEVYIKSTETGQYLAM
DTDGLLYGSQTPNEECLFLERLEENHYNTYISKKHAEKNWFVGLKKNGSCKRGPRTHYGQ
KAILFLPLPVS
>Set1-Plane1
YKKPKLLYCSNGGHFLRILPDGTVDGTRDRSDQHIQLQLSAESVGEVYIKSTETGQYLAM
DTDGLLYGSQTPNEECLFLERLEENHYNTYISKKHAEKNWFVGLKKNGSCKRGPRTHYGQ
KAILFLPLPVS
>Set1-Plane2
YKKPKLLYCSNGGHFLRILPDGTVDGTRDRSDQHIQLQLSAESVGEVYIKSTETGQYLAM
DTDGLLYGSQTPNEECLFLERLEENHYNTYISKKHAEKNWFVGLKKNGSCKRGPRTHYGQ
KAILFLPLPVS
>Set1-Plane3
YKKPKLLYCSNGGHFLRILPDGTVDGTRDRSDQHIQLQLSAESVGEVYIKSTETGQYLAM
DTDGLLYGSQTPNEECLFLERLEENHYNTYISKKHAEKNWFVGLKKNGSCKRGPRTHYGQ
KAILFLPLPVS
```

Sets of four color-coded sequences are listed in this file. The missing residues are shown by dashes (none in this example). The eight-color sequences are the results divided into eight portions with a set of three cutting surfaces (CSs). The three two-color sequences are the results divided into two parts with one of the three CSs. It should be noted that the result changes every time. The number of each portion and the color relationship between the eight-color and the three two-color sequences are shown in ColorDistribution.txt.

	A	B	C	D	E	F	G	H	I	J
1	Set	red	green	cyan	magenta	yellow	purple	greenblue	blue	%CV
2	1	17	17	16	16	17	16	16	16	2.96
3	2	15	17	16	17	17	17	17	15	5.23
4	3	16	17	15	17	17	15	18	16	6.06
5	4	17	17	15	18	16	15	16	17	6.06
6	5	16	17	16	16	17	18	17	14	6.78
7	6	18	17	16	16	16	14	17	17	6.78
8	7	16	16	15	19	17	18	15	15	8.6
9	8	15	19	17	14	16	16	16	18	9.13
10	9	17	14	16	19	18	14	16	17	10.1
11	10	14	19	16	15	15	16	18	18	10.1
12	11	15	16	16	19	18	18	13	16	10.98
13	12	18	14	17	17	18	12	16	19	13.29
14										
15	Set	PL1		PL2		PL3		%CV		
16		red	green	red	green	red	green			
17	1	66	65	67	64	66	65	1.46		
18	2	65	66	66	65	65	66	0.76		
19	3	66	65	65	66	65	66	0.76		
20	4	64	67	65	66	67	64	1.92		
21	5	66	65	68	63	65	66	2.29		
22	6	67	64	65	66	67	64	1.92		
23	7	63	68	67	64	66	65	2.61		
24	8	64	67	66	65	65	66	1.46		
25	9	67	64	63	68	66	65	2.61		
26	10	63	68	64	67	64	67	2.89		
27	11	62	69	67	64	66	65	3.39		
28	12	69	62	62	69	66	65	4.39		

32	Color	Plane1	Plane2	Plane3
33	red	red	red	red
34	green	green	red	red
35	cyan	red	green	red
36	magenta	green	green	red
37	yellow	red	red	green
38	purple	green	red	green
39	greenblue	red	green	green
40	blue	green	green	green

For example, the colors of cyan residues in the SetX-Fusion are red, green and red in SetX-Plane1, SetX-Plane2 and SetX-Plane3, respectively. The numbers of cyan residues in Set1-Fusion and red residues in Set1-Plane2 are 15 and 65, respectively.

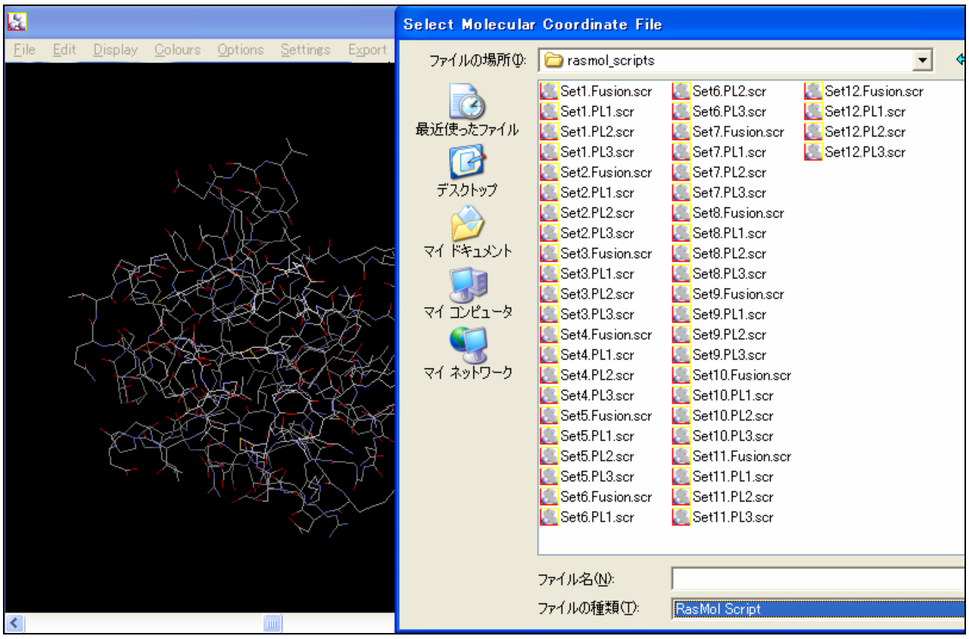

Next, open a file named target.pdb with RasMol, and then open the Set1.Fustion.scr in a rasmol_scripts directory from the RasMol menu, and an eight-colored structure is displayed.

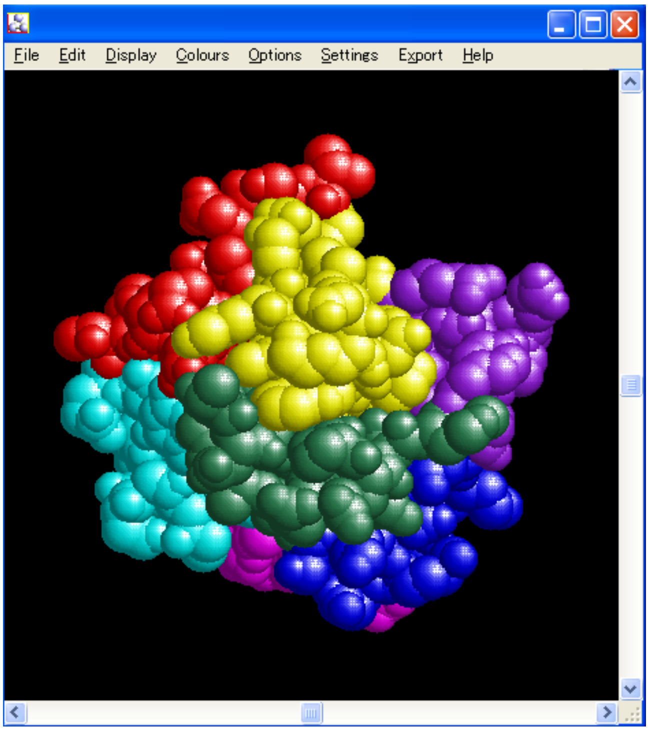

If you select Set1.PL1.scr, two-colored structure is shown. The file names correspond to the sequence names listed in the Separate.rtf. The colors of residues in each structure also correspond to that in each sequence. Next, type a command as follows.

> perl ChimeraColor.pl -D 1EVT_A.dssp.out -o FGF -c A -a 30 -r cartoon

Then, open the Set1.Fusion.scr as descried above.

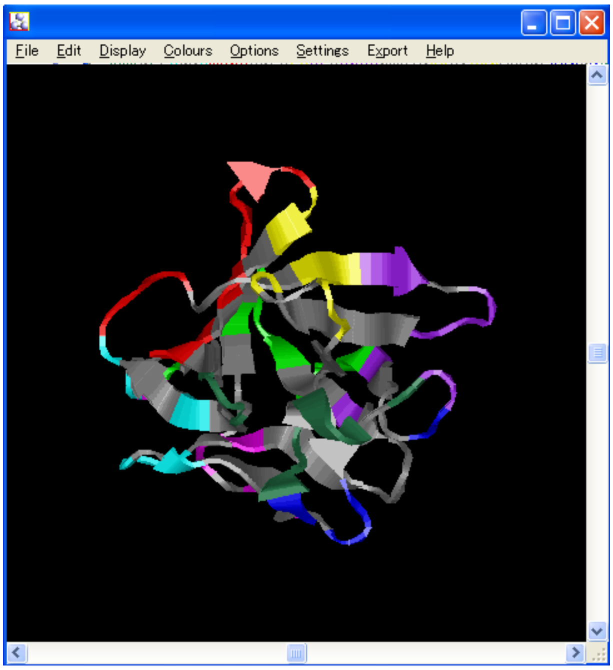

The residues that the accessible surface area (ASA) is less than 30 Å² are colored gray. Several display styles, e.g. wireframe, backbone, ribbons, strands etc., can be specified by the r option. The residues in the sequences listed in the Separate.rtf are also colored gray.

```
>Set1-Fusion
YKKPKLLYCSNGGHFLRILPDGTVDGTRDRSDQHIQLQLSAESVGEVYIKSTETGQYLAM
DTDGLLYGSQTPNEECLFLERLEENHYNTYISKKHAEKNWFVGLKKNGSCKRGPRTHYGQ
KAILFLPLPVS
>Set1-Plane1
YKKPKLLYCSNGGHFLRILPDGTVDGTRDRSDQHIQLQLSAESVGEVYIKSTETGQYLAM
DTDGLLYGSQTPNEECLFLERLEENHYNTYISKKHAEKNWFVGLKKNGSCKRGPRTHYGQ
KAILFLPLPVS
>Set1-Plane2
YKKPKLLYCSNGGHFLRILPDGTVDGTRDRSDQHIQLQLSAESVGEVYIKSTETGQYLAM
DTDGLLYGSQTPNEECLFLERLEENHYNTYISKKHAEKNWFVGLKKNGSCKRGPRTHYGQ
KAILFLPLPVS
>Set1-Plane3
YKKPKLLYCSNGGHFLRILPDGTVDGTRDRSDQHIQLQLSAESVGEVYIKSTETGQYLAM
DTDGLLYGSQTPNEECLFLERLEENHYNTYISKKHAEKNWFVGLKKNGSCKRGPRTHYGQ
KAILFLPLPVS
```

Next, type a command as follows.

> perl TCP.pl -p 1EVT.pdb -o FGFR -c C -d yes -s 147 -e 245

Then, open a target.pdb and a Set1.Fusion.scr in the FGFR directory as descried above.

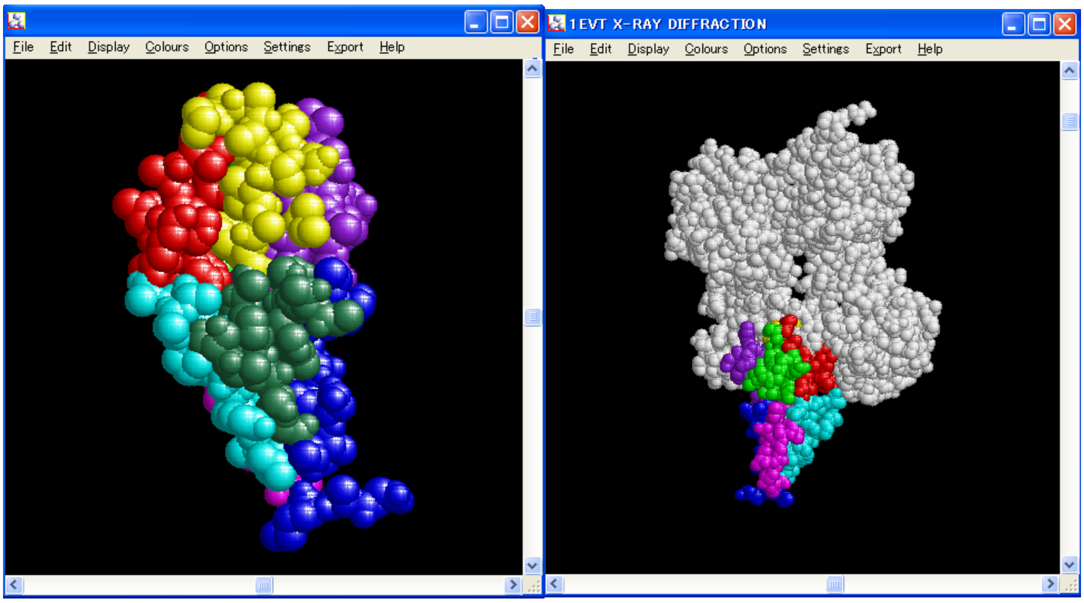

The first domain of 1EVT:C (the domain II of human FGFR1) is shown. If you open the 1EVT.pdb instead of the target.pdb, the structures other than the target region are also displayed. Although the target region can be specified by the s and the e options, the numbers are the positions described in the PDB file.

Setting up the web program

1. Perl, PHP and Apache should be set up for the server and Chime plug-in for the client based on the instructions for each program. One important configuration is register_globals = On in a php.ini file.
2. Create a directory named "TCP" and copy the four files (index.php, help.html, SearchSurface.pl, and ChimeraColor.pl) into the directory. After that, set the permissions of the TCP directory and the files to 755.
3. Create a directory named "temp" in the TCP directory and set the permission to 777.
4. Access the web interface (http://<your server name>/TCP/index.php) and confirm the top page is properly displayed.

Tutorial on the web program

We make a short tutorial and the tutorial is explained using a Windows PC. First of all,

access the TCP interface from your web browser.

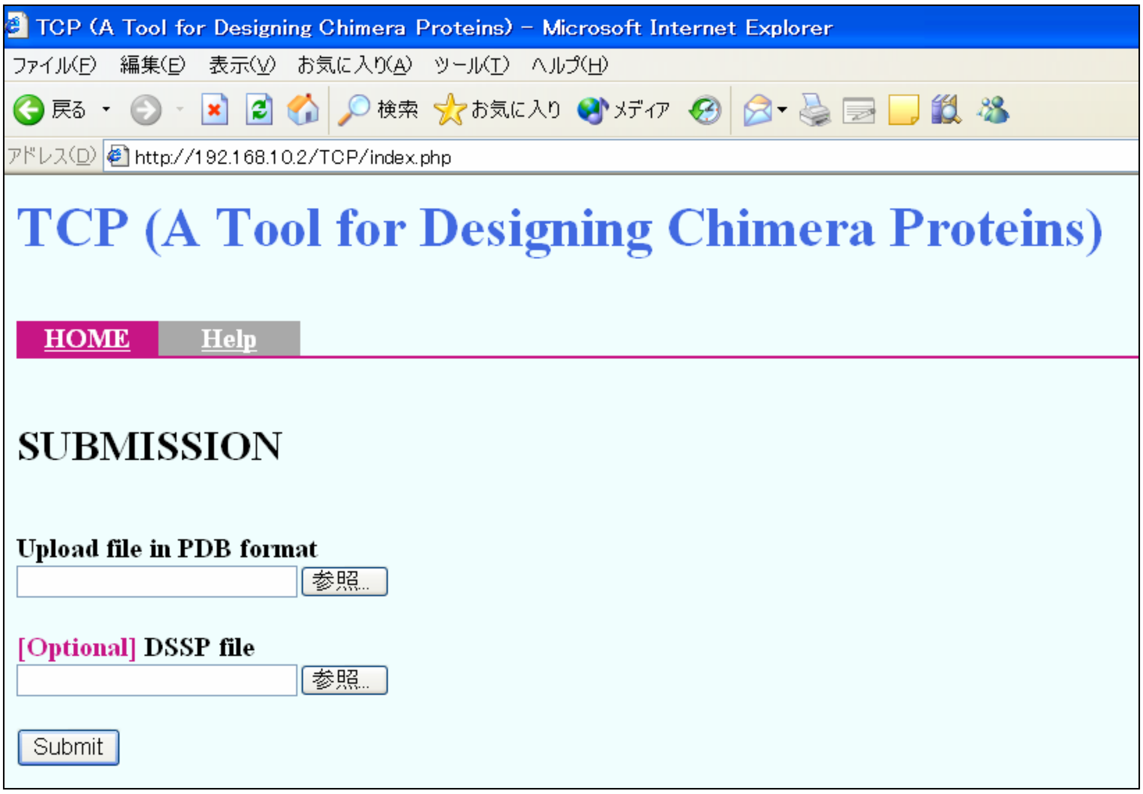

Specify 1EVT.pdb as a PDB file and 1EVT_A.dssp.out as a DSSP file, and click the submit button. A parameter setup form and 3D structures of human FGF1 and human FGFR1 will be shown.

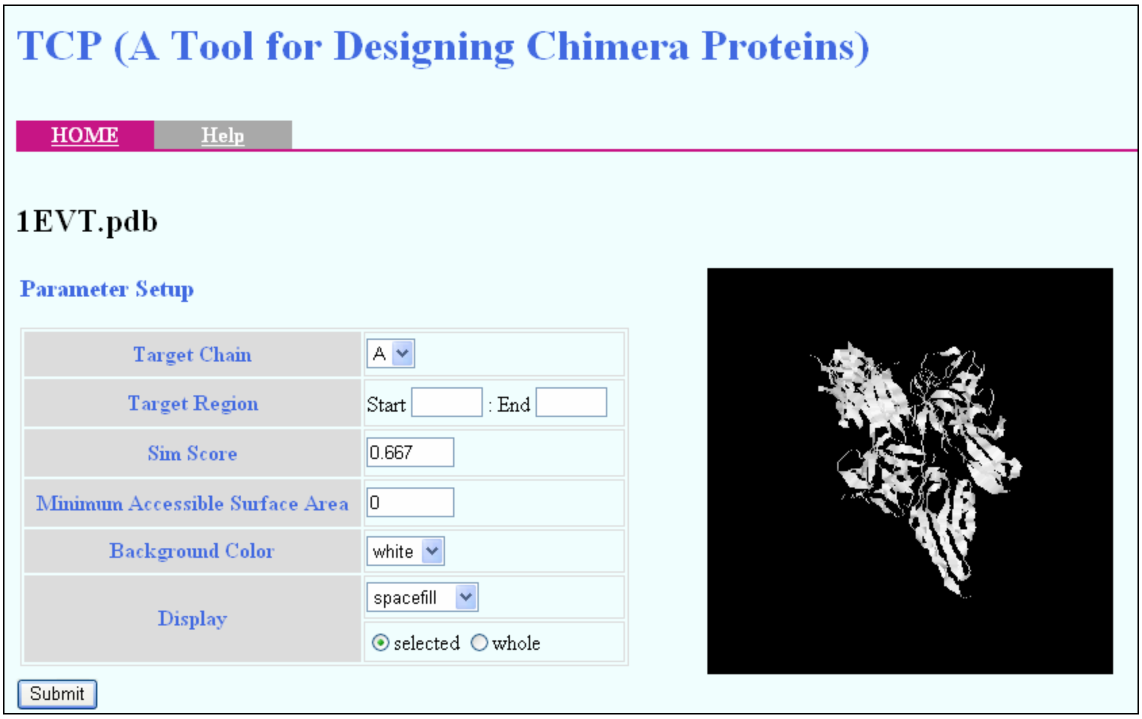

The target chain, the target region, the *Sim* score, the minimum accessible surface area, the background color and the display type can be specified here. If the DSSP file is not uploaded, the Minimum Accessible Surface Area is not shown. Select the display type "selected" and click the submit button.

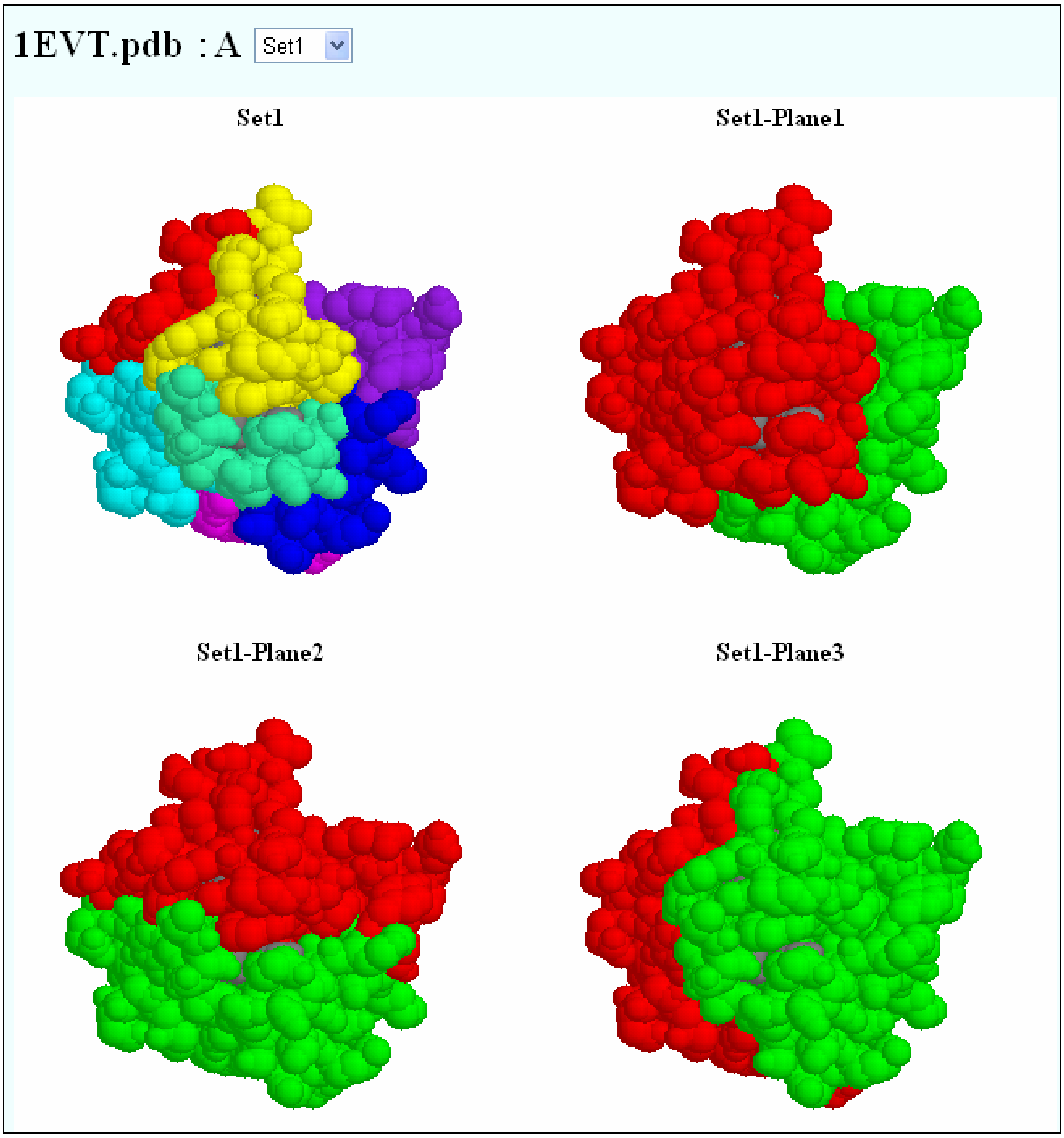

Four color-coded structures are shown in the upper part of the page. In the middle part, color-coded sequences, the centroid of the alpha-carbons of the target region, the normal vectors for the CSs and the number of residues in each part divided by each CS or the combination of three CSs are displayed. The information of the color relationships between the SetX and the SetX-PlaneY is available by clicking the help tab.

```
>Set1-Fusion
YKKPKLLYCSNGGHFLRILPDGTVDGTRDRSDQHIQLQLSAESVGEVYIKSTETGQYLAMDTDGLLYGSQTPNEECLFLE
RLEENHYNTYISKKHAEKNWFVGLKKNGSCKRGPRTHYGQKAILFLPLPVS
>Set1-Plane1
YKKPKLLYCSNGGHFLRILPDGTVDGTRDRSDQHIQLQLSAESVGEVYIKSTETGQYLAMDTDGLLYGSQTPNEECLFLE
RLEENHYNTYISKKHAEKNWFVGLKKNGSCKRGPRTHYGQKAILFLPLPVS
>Set1-Plane2
YKKPKLLYCSNGGHFLRILPDGTVDGTRDRSDQHIQLQLSAESVGEVYIKSTETGQYLAMDTDGLLYGSQTPNEECLFLE
RLEENHYNTYISKKHAEKNWFVGLKKNGSCKRGPRTHYGQKAILFLPLPVS
>Set1-Plane3
YKKPKLLYCSNGGHFLRILPDGTVDGTRDRSDQHIQLQLSAESVGEVYIKSTETGQYLAMDTDGLLYGSQTPNEECLFLE
RLEENHYNTYISKKHAEKNWFVGLKKNGSCKRGPRTHYGQKAILFLPLPVS
```

Set	vector	x	y	z
	Centroid	-8.1	63.3	-0.2
1	1	0.7093	0.2719	0.6502
1	2	-0.2023	0.9623	-0.1816
1	3	0.6751	0.0026	-0.7376

Set	red	green	cyan	magenta	yellow	purple	greenblue	blue	%CV
1	16	16	17	17	17	16	15	17	4.25
2	17	18	16	15	16	15	17	17	6.06
3	17	17	15	16	16	15	17	18	6.06
4	14	17	18	17	17	17	15	16	7.44
5	14	16	16	18	17	16	18	16	7.44
6	18	17	15	16	18	14	17	16	8.04
7	17	15	16	19	16	15	15	18	8.6
8	17	16	17	18	16	17	13	17	8.6
9	15	17	18	16	14	16	19	16	9.13
10	18	16	17	17	17	18	15	13	9.63
11	14	19	16	15	15	15	18	19	11.4

Set	PL1		PL2		PL3		%CV
	red	green	red	green	red	green	
1	65	66	65	66	66	65	0.76
2	66	65	66	65	66	65	0.76
3	65	66	65	66	65	66	0.76
4	64	67	65	66	66	65	1.46
5	65	66	63	68	64	67	2.61
6	68	63	67	64	66	65	2.61
7	64	67	63	68	67	64	2.89
8	63	68	66	65	68	63	3.15
9	66	65	62	69	66	65	3.15
10	67	64	69	62	68	63	4.02
11	63	68	63	68	64	67	3.39

The CS sets can be changed by the pulldown menu at the top of this page, and you can select the favorable CS sets. An input form to change the parameters is shown in the lower part.

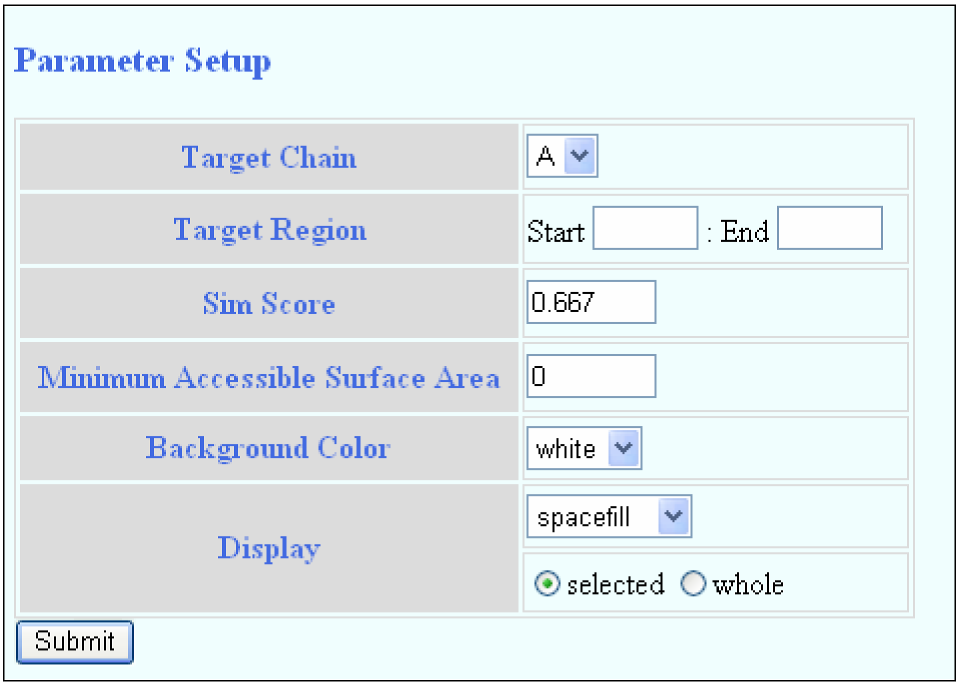

If you change the target chain and/or the target region and/or the *Sim* score, the program searches the CS sets again. If you change only the minimum accessible surface area and/or the background color and/or the display, the program uses the existing CS sets. Please compare the results by changing the parameters.

References

1. http://www.perl.org/
2. http://www.activestate.com/
3. http://www.umass.edu/microbio/rasmol/
4. http://www.mdl.com/
5. http://swift.cmbi.ru.nl/gv/dssp/
6. http://bioweb.pasteur.fr/seqanal/interfaces/dssp-simple.html
